# Reversibility of Cardiac Involvement in Acromegaly Patients After Surgery: 12-Month Follow-up Using Cardiovascular Magnetic Resonance

**DOI:** 10.3389/fendo.2020.598948

**Published:** 2020-10-21

**Authors:** Xiaopeng Guo, Yihan Cao, Jian Cao, Xiao Li, Peijun Liu, Zihao Wang, Lu Gao, Xinjie Bao, Bing Xing, Yining Wang

**Affiliations:** ^1^Neurosurgery, Peking Union Medical College Hospital, Chinese Academy of Medical Sciences and Peking Union Medical College, Beijing, China; ^2^Key Laboratory of Endocrinology of the Ministry of Health, Peking Union Medical College Hospital, Chinese Academy of Medical Sciences and Peking Union Medical College, Beijing, China; ^3^China Pituitary Disease Registry Centre, Beijing, China; ^4^China Pituitary Adenoma Specialist Council, Beijing, China; ^5^Radiology, Peking Union Medical College Hospital, Chinese Academy of Medical Sciences and Peking Union Medical College, Beijing, China

**Keywords:** acromegaly, cardiac reversibility, cardiovascular magnetic resonance, endocrine remission, gender

## Abstract

**Purpose:**

Cardiac comorbidity is one of the leading causes of death among acromegaly patients. We aimed to investigate the reversibility of acromegalic cardiac involvement after surgical treatment using the gold standard method, cardiovascular magnetic resonance, and to explore the effects of endocrine remission and gender on reversibility.

**Methods:**

In this single-center, prospective cohort study, fifty untreated acromegaly patients were enrolled. Comprehensive cardiac assessments were performed using a 3.0 T magnetic resonance scanner before and 3 and 12 months after transsphenoidal adenomectomy.

**Results:**

Preoperatively, left ventricular (LV) enlargement (13.0%), LV systolic dysfunction (6.5%), right ventricular (RV) enlargement (4.3%), RV systolic dysfunction (2.2%) and myocardial fibrosis (12.0%) were identified. On average, the LV and RV ejection fractions of acromegaly patients were higher than the healthy reference values. Male patients had thicker LV myocardia, wider ventricular diameters and more dilated pulmonary artery roots than female patients. After surgery, LV myocardial hypertrophy was reversed, the left atrium was remodeled, and ventricular systolic dysfunction recovered to normal. Cardiac alterations were detected early in the 3^rd^ postoperative month and persisted until the 12^th^ month. The interventricular septum was initially thickened in the 3^rd^ postoperative month and then recovered at the 12th month. Notable postoperative cardiac reversibility was observed in male patients but did not occur in all female patients. Patients achieving endocrine remission with normalized hormone levels had thinner LV myocardia than patients without normalized hormone levels.

**Conclusion:**

Our findings demonstrated that some of the cardiac involvement in acromegaly patients is reversible after surgical treatment which lowers hormone levels. Endocrine remission and gender significantly impacted postoperative cardiac reversibility.

## Introduction

Elevated levels of serum growth hormone (GH), combined with its product hormone insulin-like growth factor 1 (IGF-1), contribute to the systemic complications responsible for the increased mortality of acromegaly patients (1, 2). Cardiovascular comorbidity is the most common comorbidity and represents one of the most important causes of death in acromegaly patients (3–6). Myocardial hypertrophy, cardiac chamber enlargement, and diastolic and systolic dysfunction have been indicated to be common presentations of cardiac involvement in acromegaly patients (1, 5). Transsphenoidal adenomectomy and medical treatment with somatostatin analogs are the first-line treatments for acromegaly patients (2, 7, 8). Based on echocardiography, cardiac abnormalities are reversible after treatment, especially in young patients with a short disease duration upon GH and IGF-1 remission (9–12), while these abnormalities rarely improve in patients with uncontrolled GH and IGF-1 levels (13).

An accurate and comprehensive evaluation of cardiac performance is essential for the diagnosis and treatment of cardiac comorbidity (14). Although echocardiography is widely used and well studied in cardiac evaluations of acromegaly patients, it relies heavily on the experience of the operator and suitable echocardiographic windows (15). Evaluations of the heart with the gold standard cardiovascular magnetic resonance (CMR) offer objective and reproducible results, and CMR can precisely assess cardiac structure and function and even evaluate parameters that are difficult to precisely assess by echocardiography, such as myocardial fibrosis and ventricular volumes (16–19). Several studies have used CMR to evaluate cardiac involvement in acromegaly patients (20–25), and the results of these studies showed that the incidence rates of myocardial fibrosis, left ventricular (LV) systolic dysfunction (LVSD), and LV hypertrophy in acromegaly patients were 0% to 14.8%, 0% to 12.5%, and 5% to 72%, respectively. Some of these studies focused on the posttreatment reversibility of cardiac involvement, but the results are controversial. Bogazzi et al. (22) demonstrated that acromegaly patients with controlled disease after using somatostatin analogs had a greater reduction in the LV mass index than those without controlled disease. dos Santos Silva et al. (23) found no clinically relevant cardiac improvements after medical therapy with octreotide. Andreassen et al. (21), however, reported a decrease in cardiac function 3 months after treatment. Compared to medical treatment, transsphenoidal adenomectomy allows GH to decline drastically and immediately after tumor removal. The reversibility of cardiac involvement after surgery has not been systematically evaluated in acromegaly patients by CMR, and we propose that their postoperative cardiac reversibility may differ from that in patients who received medical treatment in the literature.

On the basis of our previous study on baseline cardiac involvement in a series of untreated Chinese acromegaly patients using CMR (25), the current study focused on the reversibility of cardiac involvement after transsphenoidal adenomectomy and the impacts of gender and endocrine remission (ER). Here, we applied CMR to 50 untreated acromegaly patients and analyzed the comprehensive qualitative and quantitative CMR data before and after surgery to test our hypothesis that cardiac involvement in acromegaly patients is reversible following successful surgical treatment and that gender and ER influence postoperative cardiac reversibility.

## Materials and Methods

### Patient Population

Untreated acromegaly patients were enrolled in this single-center, prospective, cohort study. This study was performed in accordance with the Declaration of Helsinki and was approved by the Institutional Review Board at PUMCH (No. ZS-1293). Written informed consent was obtained from all patients.

The inclusion criteria were as follows: 1) an IGF-1 level exceeding the age- and gender-matched reference range and a GH level exceeding 1.0 ng/ml following an oral glucose tolerance test (OGTT) (3); 2) a pituitary tumor identified on sellar magnetic resonance imaging (MRI) (3, 26); 3) adult age, with no restrictions on gender; 4) typical clinical manifestations of acromegaly; and 5) normal levels of other pituitary-related hormones, including in the patients on hormone replacement therapy, to eliminate the potential effects of hypopituitarism on the heart. The exclusion criteria were as follows: 1) hepatic disease, renal disease, implanted metal or claustrophobia; 2) recurrent pituitary adenomas; and 3) known primary cardiovascular diseases before the onset of acromegaly. Inappropriate use of the reference ranges might lead to improper data interpretation (23). Therefore, we referred to the article by Le et al. (18) as the control reference. The authors enrolled 180 healthy Chinese volunteers and grouped them according to gender and age in 10-year intervals. The mean values and upper and lower limits of the CMR parameters in each subgroup were given in that study.

### Study Design and Follow-up

Acromegaly patients were clinically diagnosed, and CMR was performed before surgery. Clinical data (gender, age, body mass index (BMI) and disease duration) and hormone levels (random GH, GH nadir and fasting IGF-1) were recorded. The disease duration was defined as the duration from the onset of acromegaly symptoms until the clinical diagnosis. Microscopic transsphenoidal adenomectomy was performed for all patients, and the pathology of GH-secreting pituitary adenomas was confirmed.

The patients were re-evaluated 3 months and 12 months after transsphenoidal surgery. CMR scans were performed, and hormone levels were measured during the follow-up period. ER was defined as a random GH<1.0 ng/ml or a GH nadir<0.4 and age-gender normalized IGF-1 (27). The IGF-1 reference range used in this study referred to our published article (28). Repeated surgery, octreotide LAR treatment and observation were recommended for recurrent patients according to the radiological findings and hormone levels.

### CMR Image Acquisition

CMR was performed on a 3.0T superconducting MR scanner (Siemens Healthineers, Germany). Cine images were acquired with an electrocardiogram-gated two-dimensional balanced steady-state free precession sequence during multiple breath holds. Two-, 3- and 4-chamber long-axis and short-axis slices were acquired. The key parameters were as follows: repetition time/echo time, 3.3/1.43 msec; flip angle, 55°–70°; voxel size, 1.6 × 1.6 × 6.0 mm; temporal resolution, 45.6 msec; and bandwidth, 962 Hz/pixel. A bolus of gadolinium (0.5 mmol/ml, Beijing BEILU Pharmaceutical Co., Ltd.) was injected at a dose of 0.05 mmol/kg and a flow rate of 4 ml/s for first-pass perfusion imaging. Another bolus of gadolinium at a dose of 0.1 mmol/kg and a flow rate of 1 ml/s was then given, followed by late gadolinium enhancement (LGE) imaging 10 to 15 min later. LGE images were acquired with a 2D phase-sensitive inversion-recovery gradient-echo pulse sequence. Focal myocardial fibrosis was identified by the presence of LGE on a special focus shown on both short- and long-axis stacks.

### CMR Parameter Extraction and Cardiac Abnormality Identification

**Figure 1:**
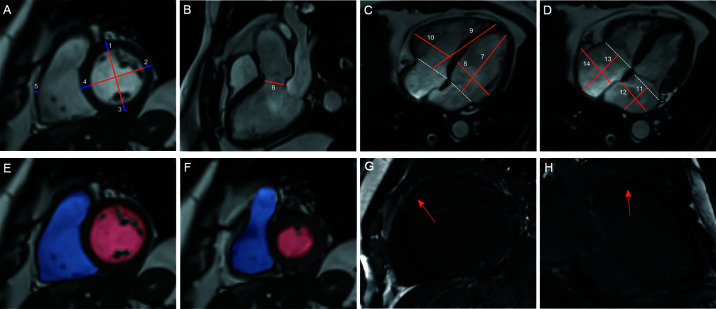
Protocol for CMR contouring of the ventricles, atria and artery roots in acromegaly patients. Left ventricular (*LV*) anterior wall thickness (1), LV lateral wall thickness (2), LV posterior wall thickness (3), interventricular septum thickness (4) and right ventricular (*RV*) lateral wall thickness (5) were measured on the short-axis slice **(A)**. The LV outflow tract diameter (6) was measured on the 3-chamber long-axis slice **(B)**. The LV longitudinal diameter (*LVLD*) (7), LV transverse diameter (*LVTD*) (8), RVLD (9) and RVTD (10) were measured on the 4-chamber long-axis slice in the LV end-diastolic phase **(C)**. The left atrial (*LA*) longitudinal diameter (*LALD*) (11), LA transverse diameter (*LATD*) (12), right atrial (*RA*) longitudinal diameter (*RALD*) (13) and RA transverse diameter (*RATD*) (14) were measured on the 4-chamber long-axis slice in the LV end-systolic phase **(D)**. LV and RV volumes were contoured slice by slice on the short-axis stacks in diastole **(E)** and systole **(F)**. Papillary muscles were excluded from the LV volume evaluation but included in the RV volume evaluation. Late gadolinium enhancement (*LGE*) was identified on both short-axis and long-axis stacks **(G**, **H)**.

CMR images were transmitted into Circle Cardiovascular Imaging software (version 5.3, Canada). **Figure 1** shows the protocol for CMR analysis. For ventricular wall thickness, we evaluated LV anterior wall (LVAW) thickness, LV lateral wall thickness, LV posterior wall thickness, interventricular septum (IVS) thickness and right ventricular (RV) lateral wall thickness. For dimensions of the heart and arteries, we evaluated the LV longitudinal diameter (LVLD), LV transverse diameter (LVTD), RVLD, RVTD, left atrial LD (LALD), LATD, right atrial LD (RALD), and RATD. The pulmonary artery root diameter (PARD) and LV outflow tract diameter (LVOTD) were measured on the 3-chamber long-axis slice. For ventricular volume, we evaluated the indexed LV end-diastolic volume (LVEDV), indexed LV end-systolic volume (LVESV), and indexed RVEDV and RVESV. The LV ejection fraction (LVEF) and RVEF were calculated as the reduction in ventricular volume from the EDV to the ESV divided by the EDV.

The reference ranges for ventricular volume and systolic function in Le’s study were used to identify qualitative cardiac abnormalities by transforming the quantitative values in this study into z scores (18). Therefore, the LVEDV, RVEDV, LVEF and RVEF values were transformed accordingly. A z score >2 standard deviations (SDs) or <−2 SDs was recognized as abnormal.

### Hormone Assays and Sellar MRI

Blood samples were collected in the morning after an 8-h fasting period for hormone analysis. Chemiluminescence assays (Siemens Healthcare Diagnostics Products Ltd., UK) were used to measure fasting GH and IGF-1 levels. GH levels were also measured at 30, 60, 120, and 180 min after an OGTT. Contrast-enhanced sellar MRI (Discovery MR750, GE, USA) was performed. The typical radiological imaging features of a pituitary adenoma include a solid hypo/isointense mass on T1-weighted imaging, a hyper/isointense mass on T2-weighted imaging and reduced enhancement after gadolinium administration.

### Statistical Analysis

SPSS (IBM SPSS Statistics, version 23.0, USA) was used to analyze the data. GraphPad Prism (GraphPad Software, version 8.1, USA) was used to generate bar charts. Categorical variables are presented as numbers and percentages. Continuous values are presented as the means ± SDs. Student’s t-test or the Mann-Whitney U test was used to compare continuous data according to the data distribution. The χ^2^ test was used to analyze correlations among categorical variables. Statistical significance was defined as *p*<0.05.

## Results

### Characterization of the Study Population

The baseline clinical characteristics and hormone levels of the 50 untreated acromegaly patients, including 27 males and 23 females, are listed in **Table 1**. No differences in age or gender were found between the acromegaly patients and healthy controls. Seven patients had hypertension at diagnosis, and their arterial blood pressure was controlled normal using antihypertensive drugs. Diabetes mellitus (DM) was diagnosed in 6 patients. Insulin was subcutaneously injected during the perioperative period, and the fasting and postprandial blood glucose levels were controlled within the normal range.

**Table 1:** CMR parameters and clinical characteristics of acromegaly patients before surgery and comparisons with the Chinese reference values.

	Acromegaly patients, n = 50
Cardiovascular wall thickness and ventricular mass	
LV anterior wall thickness, mm^a^	7.1 ± 2.2 (2.3–14.7)
LV lateral wall thickness, mm^a^	6.9 ± 1.7 (3.7–10.5)
LV posterior wall thickness, mm^a^	7.0 ± 1.5 (4.6–11.2)
Interventricular septum thickness, mm^a^	9.3 ± 2.3 (5.9–16.4)
RV lateral wall thickness, mm	2.8 ± 1.1 (1.3–5.4)
LV mass (with papillary muscles), g	104 ± 32**
Indexed LV mass, g/m^2^	56 ± 15**
Dimensions of the atriums, ventricles and artery root^a^	
LV longitudinal diameter, cm	9.3 ± 1.0 (5.2–11.3)
LV transverse diameter, cm	5.0 ± 0.4 (4.0–6.2)
RV longitudinal diameter, cm	8.8 ± 0.9 (5.1–11.5)
RV transverse diameter, cm	4.3 ± 0.5 (3.2–5.8)
LA longitudinal diameter, cm	3.2 ± 0.5 (2.2–4.1)
LA transverse diameter, cm	4.8 ± 0.7 (3.5–7.5)
RA longitudinal diameter, cm	4.7 ± 0.6 (3.2–6.3)
RA transverse diameter, cm	4.4 ± 0.6 (3.2–5.7)
Pulmonary artery root diameter, cm	2.6 ± 0.4 (1.7–3.6)
LV outflow tract, cm	3.0 ± 0.5 (2.3–4.5)
Ventricular volume and systolic function	
Indexed LV end-diastolic volume, ml/m^2^	79.2 ± 13.7 (51.1–108.6)*
Indexed LV end-systolic volume, ml/m^2^	30.1 ± 6.9 (18.4–45.0)
Indexed RV end-diastolic volume, ml/m^2^	83.1 ± 16.3 (44.9–119.0)
Indexed RV end-systolic volume, ml/m^2^	35.7 ± 10.9 (17.0–61.8)*
LV ejection fraction, %	62.0 ± 5.8 (48.3–76.1)*
RV ejection fraction, %	57.4 ± 7.3 (38.3–77.9)**
Myocardial late gadolinium enhancement, n/%^a^	6/12.0%
Clinical characteristics	
Age, years	41.6 ± 12.8 (18–73)
Male, n/%	27/54.0%
Body mass index, kg/m^2^	26.0 ± 3.6 (20.5–40.8)**
Disease duration, months	60.0 (5–240)
Fasting growth hormone, ng/ml	18.8 (4.2–242.0)
Growth hormone nadir after the OGTT, ng/ml	13.1 (1.5–219.0)
Fasting insulin-like growth factor 1, ng/ml	899.0 (394.0–1484.0)

Ranges of the CMR parameters and clinical indices are given for reference. LA, left atrial; LV, left ventricular; RA, right atrial; RV, right ventricular; OGTT, oral glucose tolerance test.

*(p < 0.05) and **(p < 0.01) indicate significant differences in CMR parameters between the acromegaly patients and the healthy Chinese controls.

a indicates that no reference values are available.

Forty-five patients (23 males and 22 females) completed the 3-month follow-up, 24 of whom (53.3%) reached ER. Among the 21 patients without ER, 12 continued observation, 5 underwent repeated surgery, and 4 received octreotide LAR treatment. Thirty-five patients (18 males and 17 females) completed the 12-month follow-up, 25 (71.4%) of whom reached ER. We compared the postoperative clinical characteristics, including age, gender, hypertension, DM, BMI and hormone levels, of the patients with the preoperative characteristics. Apart from significantly reduced postoperative GH and IGF-1 levels, no other clinical differences were found.

Quantitative CMR parameters and LGE were evaluated in 50, 45 and 35 patients before surgery, 3 months after surgery and 12 months after surgery, respectively. However, the z scores of 4 patients before surgery, 4 patients at 3 months after surgery, and 2 patients at 12 months after surgery were not calculated because of age restrictions in Le’s study (20–69 years). Therefore, cardiac abnormalities, including ventricular systolic dysfunction (<−2 SDs) and ventricular enlargement (>2 SDs), were evaluated in 46, 41 and 33 patients before surgery, 3 months after surgery and 12 months after surgery, respectively.

### Baseline Cardiac Involvement in Acromegaly Patients Using CMR

Before surgery, LV systolic dysfunction (LVSD), RV systolic dysfunction (RVSD), LV enlargement and RV enlargement were detected in 3 (6.5%), 1 (2.2%), 6 (13.0%), and 2 (4.3%) acromegaly patients, respectively.

The quantitative CMR parameters of the acromegaly patients are listed in **Table 1**. Acromegaly patients had a larger LV mass and indexed LV mass than the healthy controls. The average LVEF and RVEF of the acromegaly patients were both significantly higher. The LVEDV was markedly elevated, and the RVESV was notably reduced. In the acromegaly patients, the thicknesses of the LVAW, LV lateral wall and LV posterior wall were all thinner than the IVS and thicker than the RV lateral wall, the LALD was shorter than the LATD, the RALD was longer than the RATD, and the LVOT was significantly wider than the PAR. Patients with a history of hypertension had significantly shorter LVLD and RVLD, longer LALD and RALD and larger LVOTD (all p<0.05). Patients with a history of DM had significantly thicker LVAW and LV lateral walls, lower LVEF and RVEF and larger PARD (all p<0.05). Age, BMI, and disease duration, but not smoking, GH and IGF-1, also affected CMR parameters (**Supplementary Table 1**).

Six acromegaly patients (12.0%) had LGE on CMR. Areas of LGE in all these patients were focal and located in the midmyocardium, with 3 in the IVS, 1 in both the IVS and LVAW, 1 in both the IVS and LV lateral wall, and 1 in the LV posterior wall. Patients with LGE had a thicker IVS than patients without LGE, while the thicknesses of the other ventricular walls were similar between the two groups.

### Reversibility in the Cardiac Structure and Function After Surgery

**Figure 2:**
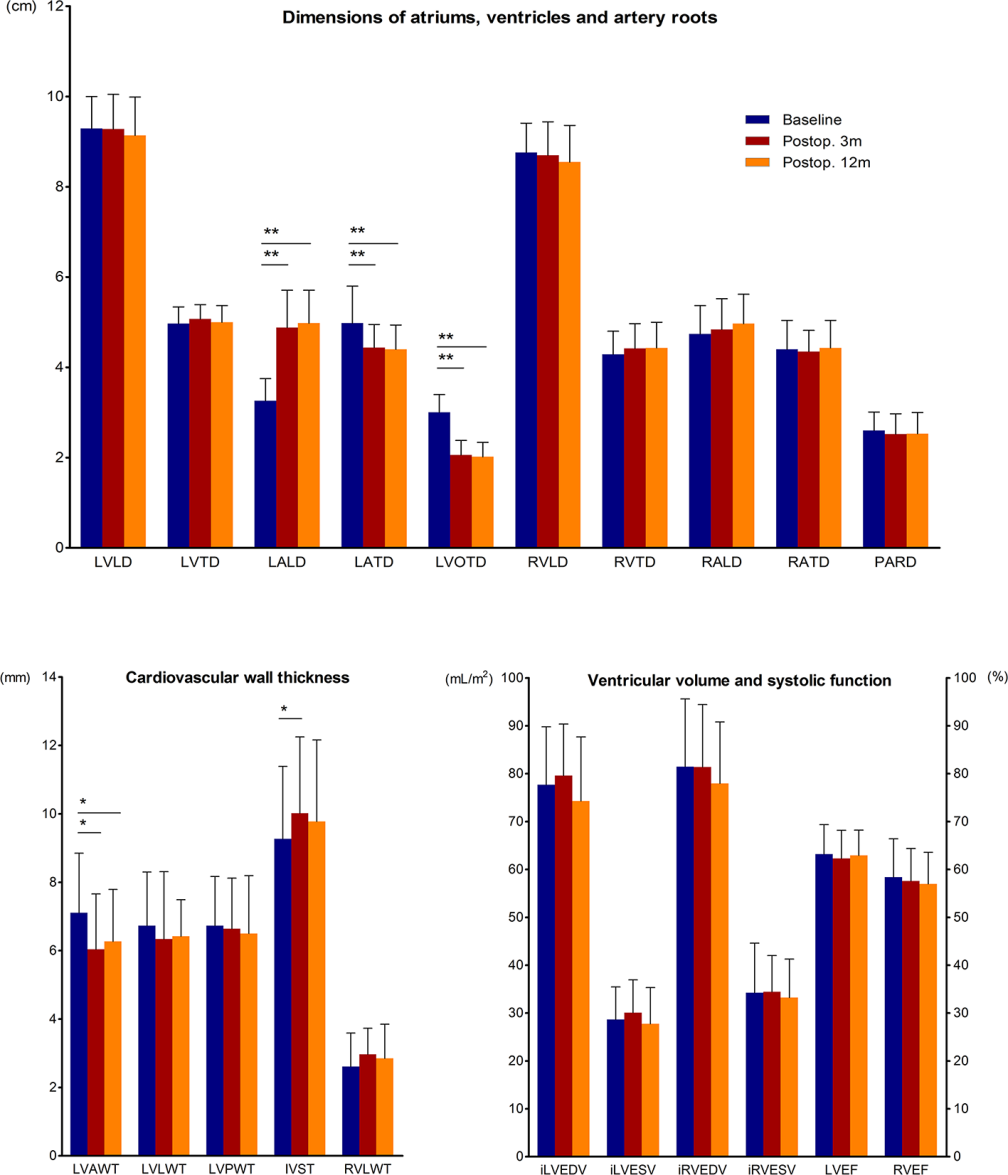
Quantitative changes in CMR parameters before and 3 and 12 months after adenomectomy. CMR parameters of acromegaly patients before and 3 and 12 months after surgery are presented as the means and SDs in the bar chart. * (p<0.05) and ** (p<0.01) indicate significant differences. *AWT*, anterior wall thickness; *EDV*, end-diastolic volume; *ESV*, end-systolic volume; *i*, indexed; *IVST*, interventricular septum thickness; *LA*, left atrium; *LD*, longitudinal diameter; *LV*, left ventricle; *LVEF*, left ventricle ejection fraction; *LVOTD*, left ventricle outflow tract diameter; *LWT*, lateral wall thickness; *PARD*, pulmonary artery root diameter; *Postop. 3m*, the 3^rd^ postoperative month; *Postop. 12m*, the 12^th^ postoperative month; *PWT*, posterior wall thickness; *RA*, right atrium; *RV*, right ventricle; *RVEF*, right ventricle ejection fraction; and *TD*, transverse diameter.

We gathered the preoperative and postoperative CMR data of acromegaly patients who completed all 3 CMR scans (**Figure 2**). The results showed that at both 3 months and 12 months after transsphenoidal surgery, the LVAW was thinned, and the LVOT was significantly narrowed. The structure of the left atrium changed dramatically in the acromegaly patients, with marked LALD shortening and LATD extension, which is defined here as “LA remodeling,” while the structures of the other heart chambers remained unchanged. The IVS was initially thickened at 3 months and then recovered to the preoperative level at 12 months after surgery.

### Effects of Gender on Postoperative Cardiac Reversibility

In male acromegaly patients, at 3 months after surgery, the LVAW was significantly thinner, the IVS was significantly thicker, the left atrium was significantly remodeled (LALD: 3.2 cm vs. 4.6 cm; LATD: 4.7 cm vs. 4.3 cm), and the LVOT was significantly narrowed (3.1 cm vs. 2.3 cm) compared to their preoperative CMR counterparts (**Table 2**). In female patients, however, the LVAW thickness change was statistically nonsignificant at this time point. At the 12^th^ postoperative month, in male acromegaly patients, in addition to the cardiac alterations detected at the 3^rd^ postoperative month, the RVLD was newly found to be elongated, and the PAR was narrowed; the IVS thickness decreased and recovered to the preoperative level. However, only LA remodeling and PAR narrowing were detected in female patients at the 12^th^ postoperative month.

**Table 2:** Reversibility of cardiac involvement in acromegaly patients after surgery, stratified by gender.

	3 months after surgery, n=45				12 months after surgery, n=35			
	Male patients, n=23		Female patients, n=22		Male patients, n=18		Female patients, n=17	
	Baseline	After surgery	Baseline	After surgery	Baseline	After surgery	Baseline	After surgery
Cardiovascular wall thickness								
LV anterior wall thickness, mm	7.6 ± 2.6	6.6 ± 1.7*	6.2 ± 1.7	5.6 ± 1.3	7.9 ± 1.6	6.7 ± 1.5**	6.5 ± 1.6	6.0 ± 1.5
LV lateral wall thickness, mm	7.1 ± 1.7	7.1 ± 2.1	6.5 ± 1.5	6.1 ± 1.4	7.5 ± 1.6	7.3 ± 1.2	6.3 ± 1.6	6.0 ± 1.1
LV posterior wall thickness, mm	7.0 ± 1.6	7.1 ± 1.5	6.6 ± 1.2	6.5 ± 1.3	7.3 ± 1.7	7.3 ± 1.6	6.6 ± 1.2	6.1 ± 1.5
Interventricular septum thickness, mm	9.5 ± 2.5	10.6 ± 2.6**	8.7 ± 1.7	9.6 ± 1.5**	10.2 ± 2.6	10.7 ± 2.3	8.7 ± 1.6	9.3 ± 2.6
RV lateral wall thickness, mm	2.8 ± 1.0	2.8 ± 0.7	2.6 ± 1.0	2.9 ± 0.8	3.1 ± 1.1	3.0 ± 1.1	2.6 ± 1.1	2.9 ± 0.9
Dimensions of the atriums, ventricles and artery root								
LV longitudinal diameter, cm	9.6 ± 1.1	9.9 ± 0.6	8.8 ± 0.6	8.8 ± 0.6	10.0 ± 0.4	9.7 ± 0.6	8.8 ± 0.4	8.6 ± 0.5
LV transverse diameter, cm	5.0 ± 0.4	5.2 ± 0.4	4.9 ± 0.4	5.0 ± 0.3	5.1 ± 0.5	5.1 ± 0.5	4.9 ± 0.4	4.9 ± 0.4
RV longitudinal diameter, cm	9.0 ± 1.1	9.2 ± 1.3	8.4 ± 0.7	8.3 ± 0.6	9.3 ± 0.5	9.7 ± 0.6	8.4 ± 0.6	8.2 ± 0.6
RV transverse diameter, cm	4.5 ± 0.5	4.7 ± 0.5	4.2 ± 0.4	4.2 ± 0.4	4.5 ± 0.6	4.7 ± 0.7	4.2 ± 0.4	4.2 ± 0.4
LA longitudinal diameter, cm	3.2 ± 0.4	4.6 ± 0.7**	3.2 ± 0.5	5.1 ± 0.8**	3.1 ± 0.5	4.9 ± 0.6**	3.4 ± 0.5	5.1 ± 0.8**
LA transverse diameter, cm	4.7 ± 0.5	4.3 ± 0.6*	5.0 ± 0.9	4.4 ± 0.5**	4.8 ± 0.5	4.4 ± 0.6*	5.1 ± 0.9	4.4 ± 0.4**
RA longitudinal diameter, cm	4.8 ± 0.7	4.9 ± 0.6	4.6 ± 0.6	4.8 ± 0.7	4.7 ± 0.7	5.1 ± 0.5*	4.7 ± 0.5	4.8 ± 0.8
RA transverse diameter, cm	4.5 ± 0.5	4.6 ± 0.4	4.3 ± 0.6	4.2 ± 0.5	4.5 ± 0.6	4.5 ± 0.4	4.3 ± 0.7	4.3 ± 0.7
LV outflow tract, cm	3.1 ± 0.5	2.3 ± 0.3**	2.9 ± 0.4	1.9 ± 0.3**	3.0 ± 0.4	2.2 ± 0.3**	3.0 ± 0.4	1.9 ± 0.2**
Pulmonary artery root diameter, cm	2.7 ± 0.3	2.6 ± 0.3	2.5 ± 0.4	2.5 ± 0.5	2.7 ± 0.4	2.5 ± 0.3*	2.6 ± 0.4	2.6 ± 0.5
Ventricular volume and systolic function								
Indexed LV end-diastolic volume, ml/m^2^	79.0 ± 12.3	81.9 ± 11.7	78.9 ± 13.2	78.5 ± 11.0	79.4 ± 12.9	77.6 ± 13.8	77.1 ± 15.0	71.7 ± 13.3
Indexed LV end-systolic volume, ml/m^2^	30.8 ± 6.1	33.1 ± 6.5	29.1 ± 7.2	28.1 ± 1.5	30.0 ± 7.3	29.2 ± 8.9	28.1 ± 7.2	26.2 ± 7.0
Indexed RV end-diastolic volume, ml/m^2^	84.9 ± 16.1	86.3 ± 12.6	80.2 ± 13.3	78.1 ± 11.6	86.0 ± 17.1	83.4 ± 15.1	78.5 ± 15.5	73.2 ± 11.7
Indexed RV end-systolic volume, ml/m^2^	37.9 ± 11.3	38.1 ± 7.9	33.1 ± 8.8	31.4 ± 6.3	37.6 ± 12.6	37.1 ± 10.9	31.6 ± 8.8	29.0 ± 5.3
LV ejection fraction, %	60.7 ± 6.6	59.7 ± 3.9	63.4 ± 4.8	64.3 ± 5.6	62.3 ± 6.8	62.9 ± 6.0	63.7 ± 5.2	63.7 ± 5.5
RV ejection fraction, %	55.7 ± 8.3	56.0 ± 5.4	59.1 ± 6.0	59.4 ± 7.1	56.8 ± 9.3	55.6 ± 7.0	60.1 ± 5.4	59.8 ± 7.0

LA, left atrial; LV, left ventricular; RA, right atrial; RV, right ventricular.

*(p < 0.05) and **(p < 0.01) indicate statistically significant differences between the baseline and postoperative CMR parameters.

### Effects of ER on Postoperative Cardiac Reversibility

Although postoperative hormone levels were not normalized in patients without ER, the reductions in these hormone indices were all significant (**Table 3**). LA remodeling and LVOT narrowing were found in both groups of patients with ER and without ER at both the 3^rd^ postoperative month and the 12^th^ postoperative month. The LVAW was notably thinned in patients with ER but not in patients without ER. The absolute reduction in the LVOTD in patients with ER was significantly greater than that in patients without ER at the 3^rd^ postoperative month.

**Table 3:** Effects of endocrine remission on postoperative cardiac reversibility.

	3 months after surgery, n=45						*p*1	12 months after surgery, n=35						*p*2
	Patients with ER, n=24			Patients without ER, n=21				Patients with ER, n=25			Patients without ER, n=10			
	Preop.	Postop.	Change	Preop.	Postop.	Change		Preop.	Postop.	Change	Preop.	Postop.	Change	
Random GH, ng/ml	23.9 ± 36.2	0.8 ± 0.7**	–	63.9 ± 64.3	11.1 ± 17.9**	–	–	26.3 ± 37.1	0.8 ± 0.6**	–	45.1 ± 35.5	3.2 ± 1.8**	–	–
GH nadir after the OGTT, ng/ml	16.3 ± 24.7	0.2 ± 0.2**	–	48.0 ± 58.0	6.1 ± 8.5**	–	–	17.0 ± 24.4	0.3 ± 0.1**	–	31.5 ± 30.0	1.4 ± 0.4**	–	–
z value for IGF-1, ng/ml	6.4 ± 1.2	0.4 ± 2.2**	–	6.7 ± 1.6	4.5 ± 1.4**	–	–	6.3 ± 0.3	0.9 ± 0.2**	–	6.9 ± 0.4	3.8 ± 0.2**	–	–
Cardiovascular wall thickness														
LV anterior wall thickness, mm	7.4 ± 1.7	6.2 ± 1.9*	−1.20	6.4 ± 2.7	6.0 ± 1.2	−0.40	0.201	7.5 ± 1.8	6.5 ± 1.6*	−1.06	6.6 ± 1.5	6.0 ± 1.3	−0.53	0.347
LV lateral wall thickness, mm	7.0 ± 1.7	6.6 ± 2.2	−0.38	6.6 ± 1.5	6.6 ± 1.3	−0.08	0.585	6.9 ± 1.6	6.6 ± 1.1	−0.31	7.0 ± 1.8	7.0 ± 1.9	0.02	0.557
LV posterior wall thickness, mm	6.9 ± 1.6	6.9 ± 1.5	0.05	6.8 ± 1.3	6.7 ± 1.3	−0.08	0.783	6.9 ± 1.5	6.7 ± 1.7	−0.22	7.0 ± 1.5	6.7 ± 1.5	−0.23	0.985
Interventricular septum thickness, mm	9.5 ± 2.4	10.3 ± 2.3*	0.76	8.6 ± 1.8	9.9 ± 2.0*	1.23	0.311	9.7 ± 2.3	10.1 ± 2.7	0.40	9.0 ± 2.4	9.9 ± 2.2	0.85	0.503
RV lateral wall thickness, mm	2.7 ± 1.2	2.8 ± 0.8	0.07	2.6 ± 0.8	2.9 ± 0.8	0.27	0.613	2.8 ± 1.1	3.0 ± 1.0	0.20	2.8 ± 1.1	2.7 ± 0.8	−0.10	0.526
Dimensions of the atriums, ventricle and artery root														
LV longitudinal diameter, cm	9.1 ± 1.1	9.3 ± 0.8	0.20	9.3 ± 0.8	9.4 ± 0.7	0.06	0.613	9.5 ± 0.8	9.2 ± 0.9	−0.26	9.2 ± 0.7	9.1 ± 0.7	0.05	0.218
LV transverse diameter, cm	4.9 ± 0.4	5.1 ± 0.3	0.18	5.0 ± 0.3	5.1 ± 0.4	0.03	0.075	5.0 ± 0.5	5.0 ± 0.4	0.03	5.1 ± 0.5	5.0 ± 0.5	−0.09	0.343
RV longitudinal diameter, cm	8.6 ± 0.9	8.8 ± 0.9	0.20	8.7 ± 1.1	8.9 ± 1.0	−0.17	0.273	8.9 ± 0.6	8.6 ± 0.9	−0.37	8.7 ± 0.9	8.7 ± 0.6	−0.01	0.147
RV transverse diameter, cm	4.3 ± 0.5	4.4 ± 0.5	0.15	4.4 ± 0.5	4.5 ± 0.6	0.10	0.695	4.3 ± 0.6	4.4 ± 0.6	0.15	4.5 ± 0.4	4.5 ± 0.6	0.02	0.516
LA longitudinal diameter, cm	3.3 ± 0.5	4.9 ± 0.9**	1.66	3.1 ± 0.5	4.7 ± 0.5**	1.60	0.755	3.2 ± 0.5	5.1 ± 0.7**	1.88	3.3 ± 0.5	4.7 ± 0.6**	1.43	0.069
LA transverse diameter, cm	5.1 ± 0.9	4.4 ± 0.5**	−0.71	4.6 ± 0.4	4.3 ± 0.5*	−0.28	0.071	5.0 ± 0.9	4.5 ± 0.5*	−0.56	4.7 ± 0.2	4.3 ± 0.4**	−0.54	0.716
RA longitudinal diameter, cm	4.9 ± 0.6	5.0 ± 0.7	0.06	4.5 ± 0.7	4.7 ± 0.5	0.22	0.237	4.7 ± 0.6	5.0 ± 0.7	0.26	4.7 ± 0.6	4.9 ± 0.5	0.23	0.909
RA transverse diameter, cm	4.4 ± 0.7	4.4 ± 0.5	−0.02	4.4 ± 0.5	4.4 ± 0.5	−0.05	0.894	4.3 ± 0.7	4.5 ± 0.6	0.12	4.5 ± 0.6	4.4 ± 0.6	−0.14	0.338
Pulmonary artery root diameter, cm	2.8 ± 0.4	2.7 ± 0.5	−0.08	2.3 ± 0.3	2.4 ± 0.3	0.05	0.149	2.7 ± 0.4	2.4 ± 0.5	−0.12	1.4 ± 0.3	2.4 ± 0.3	−0.02	0.433
LV outflow tract, cm	3.1 ± 0.4	2.1 ± 0.3**	−1.08	2.8 ± 0.5	2.1 ± 0.4**	−0.70	0.017*	3.1 ± 0.4	2.1 ± 0.3**	−1.00	2.8 ± 0.3	1.9 ± 0.3**	−0.92	0.620
Ventricular volume and systolic function														
Indexed LV end-diastolic volume, ml/m^2^	79.7 ± 14.5	81.2 ± 11.2	1.53	78.1 ± 10.4	79.2 ± 11.8	1.02	0.905	77.0 ± 14.8	74.4 ± 13.5	−2.61	81.5 ± 10.9	75.6 ± 14.8	−5.82	0.607
Indexed LV end-systolic volume, ml/m^2^	29.3 ± 7.0	30.6 ± 7.3	1.34	30.8 ± 6.3	30.7 ± 6.6	−0.09	0.476	28.6 ± 7.1	27.4 ± 7.8	−1.17	30.2 ± 7.9	28.5 ± 9.0	−1.67	0.864
Indexed RV end-diastolic volume, ml/m^2^	80.9 ± 15.8	83.9 ± 14.4	2.97	84.5 ± 13.8	80.5 ± 10.5	−3.98	0.174	78.5 ± 16.1	77.0 ± 14.6	−1.53	92.0 ± 13.9	81.9 ± 13.6	−10.09	0.138
Indexed RV end-systolic volume, ml/m^2^	33.4 ± 10.9	35.7 ± 8.7	2.30	38.1 ± 9.2	33.9 ± 6.9	−4.22	0.026	32.4 ± 11.1	32.6 ± 10.0	0.24	40.4 ± 9.8	34.5 ± 8.2	−5.98	0.100
LV ejection fraction, %	63.3 ± 5.2	62.5 ± 5.7	−0.85	60.5 ± 6.4	61.3 ± 4.8	0.82	0.329	62.7 ± 6.3	63.5 ± 5.7	0.74	63.4 ± 5.6	62.9 ± 5.9	−0.57	0.644
RV ejection fraction, %	59.2 ± 7.9	57.5 ± 6.7	−1.77	55.2 ± 6.3	58.0 ± 6.4	2.80	0.047	59.2 ± 8.4	57.7 ± 7.4	−1.49	56.4 ± 5.6	57.6 ± 7.0	1.13	0.448

ER, endocrine remission; GH, growth hormone; IGF-1, insulin-like growth factor 1; LA, left atrial; LV, left ventricular; RA, right atrial; RV, right ventricular; Preop., preoperative period; Postop., postoperative period; OGTT, oral glucose tolerance test.

p1 indicates the differences in quantitative CMR parameter changes between the patients with and without ER at 3 months after surgery, and p2 indicates the differences at 12 months after surgery.

*(p < 0.05) and **(p < 0.01) indicate statistically significant differences between preoperative parameters and postoperative parameters.

## Discussion

In this study, we systematically assessed the dimensions of the atria, ventricles and artery roots, ventricular myocardium thicknesses, ventricular volumes, cardiac systolic function, and presentations of cardiac fibrosis in acromegaly patients using CMR and longitudinally analyzed the reversibility of cardiac involvement at 3 and 12 months after transsphenoidal adenomectomy. We found that some of the cardiac involvement in acromegaly patients was reversible after surgical treatment and that gender and ER had significant impacts on postoperative cardiac reversibility.

Ventricular systolic dysfunction, which is defined as a reduced ventricular ejection fraction, has been studied in acromegaly patients using CMR (20–24). In this study, the rates of LVSD and RVSD were 6.5% and 2.2%, respectively, which are lower than previous findings. Apart from LVSD and RVSD, our results showed elevated average ejection fractions for both ventricles in acromegaly patients compared to those in healthy controls. Since the disease duration was shorter on average in this cohort than in the abovementioned CMR studies, this parameter might provide a clue regarding the entire alteration process of acromegalic cardiac function from elevated systolic function at the beginning of the disease course to reduced systolic function and even heart failure at the end of the disease course. Notably, the etiologies of elevated LVEFs and RVEFs differed. The LVEF was elevated because of an increased LVEDV and unchanged LVESV, whereas the RVEF was elevated due to a decreased RVESV and an unchanged RVEDV.

Myocardial fibrosis, which appears as LGE on CMR, occurs in ischemic coronary disease or nonischemic hypertrophic cardiomyopathy (29). The most common location of LGE in this cohort was the IVS, and the thickness of the IVS in patients with LGE was significantly greater than that in patients without LGE. LGE in acromegaly patients in this study was present only in the midmyocardium, showing different radiological features from those in patients with coronary heart diseases (30). LGE was found in 12% of the patients in this study, which is similar to other CMR studies (23, 24). However, in a study using biopsy (31), myocardial fibrosis was found in 53.7% of the cohort. Possible reasons for this discrepancy include a long disease duration of 10 years, the inclusion of patients with coronary heart disease, and limited diagnosis and treatment of acromegaly during the study period (31).

Our results showed that male acromegaly patients had thicker ventricular walls, larger ventricular chambers and wider PARs than female patients before surgical treatment. Lei et al. (32) demonstrated that healthy males had a longer LVLD than healthy females (53 mm vs. 50 mm). We found that the LVLD in male acromegaly patients was also longer than that in female patients (97 mm vs. 88 mm). Thus, the increase in LVLD in male patients was 83%, which is larger than the 76% increase among female patients. The study by Le et al. (18) showed that the average LVEF and RVEF of healthy men were lower than those of healthy women by 4% and 7%, respectively. However, in male and female acromegaly patients, these gaps were reduced to 2% and 3%, respectively. Therefore, although hormone levels and disease durations were similar between males and females in this study, cardiac chamber enlargement and ventricular systolic function increases were more obvious in male patients than in female patients.

Cardiac abnormalities in acromegaly patients were reported to improve after treatment according to echocardiography (1, 4, 5) in both humans and cats (33, 34). However, no studies have systematically focused on the reversibility of acromegalic cardiac involvement after adenomectomy using the gold standard of CMR. In this cohort, the ejection fraction of all acromegaly patients with ventricular systolic dysfunction recovered to normal during the follow-up, showing that the functional cardiac abnormalities of acromegaly patients could be reversed by postoperative hormone reduction. Additionally, the average ventricular thickness changed markedly after surgery. These results using CMR confirmed the reversibility of some of the structural and functional cardiac involvement in acromegaly patients after adenomectomy.

LV remodeling and LA enlargement are typical presentations of hypertensive heart diseases (35, 36). Similarly, LV hypertrophy in acromegaly patients, which is caused by both excessive hormones and secondary hypertension, was identified and reported to be reversible after treatment (1, 5, 37). Our results revealed LA remodeling after surgery in acromegaly patients regardless of gender and ER for the first time. Preoperatively, a short LALD and long LATD with an average ratio of 3.2:4.8 were noted in the acromegaly patients, as evidenced by a flat atrium in the anteroposterior view that differed from the right atrium. However, after surgery, an increased LALD and a reduced LATD resulted in a reversed LALD/LATD ratio of 4.8:4.3 at 3 months and 5.0:4.4 at 12 months, manifesting as a tall atrium in the anteroposterior view. We hypothesized that this change might be a specialized response of an acromegalic left atrium to a sudden and drastic postoperative hormone reduction. The distribution of hormone receptors in the heart and their roles in postoperative LA remodeling require further verification.

Little is known about the effects of tumor resection on cardiac involvement in male acromegaly patients compared to female patients. In this study, although the female patients experienced several postoperative cardiac alterations similar to those in the male patients, including LA remodeling, transient IVS thickening and LVOT narrowing, the reversibility of LVAW hypertrophy after surgery was a unique finding in male patients. Before surgery, the LVAW of the male patients was thicker than that of the female patients, while the reduction in LVAW thickness after adenomectomy in male patients was much greater. We hypothesize that estrogen and estrogen receptors may impose potential effects on the differences in cardiac reversibility between female and male patients. Further studies are needed to explore the underlying mechanisms. Additionally, we found that the current criteria for ER (27) served as a good predictor of CMR-based postoperative cardiac reversibility. LVAW reduction was marked in patients with postoperative ER but not in patients without ER. This correlation between hormone normalization and myocardial thinning verified conclusions from previous studies determined with echocardiography (13, 38) and reiterated the importance of hormone normalization to postoperative cardiac improvements.

Our study has some limitations. First, given the low incidence of acromegaly of 1.1/100,000 (2) and the strict inclusion criteria, we enrolled only 50 patients in this study, which might contribute to false-negative results. However, this study is currently among the largest of its kind, and the highly objective and reproducible assessments of CMR were able to yield reliable results with limited samples (19). Second, three articles were candidates for healthy Chinese CMR reference values for this study (18, 38, 39). Dong and Lei’s studies had smaller sample sizes than Le’s study, and their reference ranges were not given according to age, precluding our acquisition of qualitative data by converting absolute CMR values into z scores. Thus, we finally chose the reference ranges from Le’s study. Third, 5 patients and 15 patients were lost to follow-up at the 3^rd^ and 12^th^ postoperative months, respectively. We analyzed the possible reasons. Our institute is one of the largest centers for pituitary surgery in China, and patients always come from different regions of China. Some who live far away or have difficulty affording travel expenses were more likely to complete the postoperative follow-up at local medical centers instead of returning to our institute. However, we compared the general data of the acromegaly patients before and after surgery, and the results showed consistency. Thus, the loss of patients in this study had little impact on the acquisition and interpretation of our results. Fourth, detailed metabolic parameters were not acquired, cardiac comorbidities, e.g., atherosclerosis, coronaropathy and valvular insufficiency, were not estimated, and postoperative gonadal hormones were not recorded in this study, which might have potential influence on CMR findings.

In conclusion, we applied the gold standard technique, CMR, to acromegaly patients and systematically assessed the postoperative reversibility of acromegalic cardiac involvement. Using CMR, we obtained and analyzed comprehensive cardiac parameters that were difficult to precisely and objectively evaluate by echocardiography and compared them with the corresponding parameters in healthy subjects. We detected postoperative LA remodeling and improvement of cardiac systolic function and myocardium thickness and found that gender and ER significantly impacted postoperative cardiac reversibility. The application of CMR furthered our understanding of the nature and postoperative reversibility of cardiac comorbidity in acromegaly patients.

## Data Availability Statement

All datasets presented in this study are included in the article/**Supplementary Material**.

## Ethics Statement

The studies involving human participants were reviewed and approved by the Institutional Review Board at Peking Union Medical College Hospital. The patients/participants provided their written informed consent to participate in this study.

## Author Contributions

XG, YW, and BX designed the study. XG, ZW, LG, and XB enrolled the patients and completed the arrangements for CMR for all patients before, 3 months after, and 12 months after surgery. YC, JC, XL, and PL acquired the scans and analyzed and interpreted the data. XG and YC analyzed the data and created the figures. XG drafted the manuscript. YW and BX critically revised the manuscript. All authors contributed to the article and approved the submitted version.

## Funding

This work was supported by the National Natural Science Foundation of China (grant number 81471725), the Key Technologies Research and Development Program (grant number 2016YFC1300402), and Peking Union Medical College (grant number 2017-1002-02-18). The funding bodies had no role in the design of the study; the collection, analysis, and interpretation of the data; or the writing of the manuscript.

## Conflict of Interest

The authors declare that the research was conducted in the absence of any commercial or financial relationship that could be construed as a potential conflict of interest.

## Abbreviations

BMI, body mass index; CMR, cardiovascular magnetic resonance; EDV, end-diastolic volume; ESV, end-systolic volume; FA, flip angle; GH, growth hormone; IGF-1, insulin-like growth factor 1; IVS, interventricular septum; LA, left atrium/left atrial; LD, longitudinal diameter; LV, left ventricle/left ventricular; LVAW, left ventricular anterior wall; LVEF, left ventricular ejection fraction; LVOT, left ventricular outflow tract; OGTT, oral glucose tolerance test; RA, right atrium/right atrial; RV, right ventricle/right ventricular; RVEF, right ventricular ejection fraction; PAR, pulmonary artery root; SD, standard deviation; TD, transverse diameter; TE, echo time; TR, repetition time.
