# Supplementary material for: Reversibility of Cardiac Involvement in Acromegaly Patients After Surgery: 12-Month Follow-up Using Cardiovascular Magnetic Resonance
Source: Front Endocrinol (Lausanne). 2020 Oct 21;11:598948. doi: 10.3389/fendo.2020.598948 (PMC7609918; doi:10.3389/fendo.2020.598948)
Supplement: Supplementary file 1 [file Table_1.docx]

Supplementary Table 1. Clinical relevance of CMR parameters before surgery.

|  |  | Age | BMI | Disease duration | GH level | IGF-1 level | Hypertension | DM | Smoking |
| --- | --- | --- | --- | --- | --- | --- | --- | --- | --- |
| Cardiovascular wall thickness | |  |  |  |  |  |  |  |  |
|  | LV anterior wall thickness, mm | 0.478 | 0.111 | 0.434 | 0.562 | 0.778 | 0.863 | **0.021*** | 0.547 |
|  | LV lateral wall thickness, mm | 0.233 | 0.929 | 0.840 | 0.848 | 0.951 | 0.901 | **0.007**** | 0.092 |
|  | LV posterior wall thickness, mm | 0.979 | 0.886 | 0.917 | 0.595 | 0.625 | 0.973 | 0.159 | 0.412 |
|  | Interventricular septum thickness, mm | 0.599 | 0.325 | 0.618 | 0.738 | 0.462 | 0.077 | 0.106 | 0.534 |
|  | RV lateral wall thickness, mm | 0.075 | **0.025*** | 0.923 | 0.869 | 0.165 | 0.541 | 0.174 | 0.227 |
| Dimensions of atrium, ventricle and artery root | |  |  |  |  |  |  |  |  |
|  | LV longitudinal diameter, cm | 0.938 | 0.448 | 0.222 | 0.473 | 0.763 | **0.009*** | 0.637 | 0.838 |
|  | LV transverse diameter, cm | 0.363 | 0.846 | 0.591 | 0.461 | 0.517 | 0.372 | 0.967 | 0.202 |
|  | RV longitudinal diameter, cm | 0.498 | 0.966 | 0.159 | 0.225 | 0.600 | **0.031*** | 0.793 | 0.536 |
|  | RV transverse diameter, cm | 0.657 | 0.252 | 0.866 | 0.991 | 0.888 | 0.808 | 0.156 | 0.947 |
|  | LA longitudinal diameter, cm | 0.308 | 0.711 | 0.827 | 0.896 | 0.779 | **0.033*** | 0.298 | 0.616 |
|  | LA transverse diameter, cm | **0.011*** | 0.294 | 0.157 | 0.148 | 0.131 | 0.078 | 0.813 | 0.891 |
|  | RA longitudinal diameter, cm | 0.222 | 0.581 | 0.118 | 0.403 | 0.559 | **0.022*** | 0.741 | 0.907 |
|  | RA transverse diameter, cm | **0.006*** | 0.077 | 0.432 | 0.532 | 0.487 | 0.448 | 0.151 | 0.908 |
|  | LV outflow tract, cm | **0.028*** | 0.733 | 0.491 | 0.059 | 0.142 | **0.001**** | 0.212 | 0.481 |
|  | Pulmonary artery root diameter, cm | 0.989 | 0.332 | **0.034*** | 0.564 | 0.250 | 0.091 | **0.009**** | 0.354 |
| Ventricular volume and systolic function | |  |  |  |  |  |  |  |  |
|  | Indexed LV end diastolic volumn, ml/m2 | 0.805 | 0.662 | 0.424 | 0.380 | 0.488 | 0.897 | 0.312 | 0.438 |
|  | Indexed LV end systolic volumn, ml/m2 | 0.998 | 0.534 | 0.451 | 0.650 | 0.477 | 0.793 | **0.034*** | 0.818 |
|  | Indexed RV end diastolic volumn, ml/m2 | 0.643 | 0.872 | 0.588 | 0.180 | 0.339 | 0.401 | 0.071 | 0.931 |
|  | Indexed RV end systolic volumn, ml/m2 | 0.851 | 0.802 | 0.651 | 0.397 | 0.313 | 0.331 | **0.005**** | 0.589 |
|  | LV ejection fraction, % | 0.601 | 0.645 | 0.418 | 0.641 | 0.587 | 0.681 | **0.039*** | 0.168 |
|  | RV ejection fraction, % | 0.735 | 0.952 | 0.531 | 0.489 | 0.459 | 0.509 | **0.015*** | 0.403 |

* indicates p<0.05. ** means p<0.01.
